# IC Packaging Material Identification via a Hybrid Deep Learning Framework with CNN–Transformer Bidirectional Interaction

**DOI:** 10.3390/mi15030418

**Published:** 2024-03-21

**Authors:** Chengbin Zhang, Xuankai Zhou, Nian Cai, Shuai Zhou, Han Wang

**Affiliations:** 1 School of Information Engineering, Guangdong University of Technology, Guangzhou 510006, China; 2112103037@mail2.gdut.edu.cn (C.Z.); 3122002034@mail2.gdut.edu.cn (X.Z.); 2 China Electronic Product Reliability and Environmental Testing Research Institute, Guangzhou 510006, China; zhoushuai@ceprei.com; 3 School of Mechanical and Electrical Engineering, Guangdong University of Technology, Guangzhou 510006,China; wanghangood@gdut.edu.cn

**Keywords:** IC packaging material recognition, transformer, convolutional neural network, bidirectional interaction

## Abstract

With the advancement of micro- and nanomanufacturing technologies, electronic components and chips are increasingly being miniaturized. To automatically identify their packaging materials for ensuring the reliability of ICs, a hybrid deep learning framework termed as CNN–transformer interaction (CTI) model is designed on IC packaging images in this paper, in which several cascaded CTI blocks are designed to bidirectionally capture local and global features from the IC packaging image. Each CTI block involves a CNN branch with two designed convolutional neural networks (CNNs) for CNN local features and a transformer branch with two transformers for transformer global features and transformer local-window features. A bidirectional interaction mechanism is designed to interactively transfer the features in channel and spatial dimensions between the CNNs and transformers. Experimental results indicate that the hybrid framework can recognize three types of IC packaging materials with a good performance of 96.16% F1-score and 97.92% accuracy, which is superior to some existing deep learning methods.

## 1. Introduction

Nowadays, integrated circuit (IC) components and chips are widely used in micro- and nanomanufacturing, and their reliability highly influences the regular functions of the manufacturing equipment. IC packaging isolates the core circuits in the ICs from external environments to ensure IC reliable functions, which is commonly made of metal, ceramic, and plastic materials [1]. Decapsulation with preservation of the internal structures for ICs is essential for the failure analysis of IC chips [2], which is precisely performed using techniques such as nano-scale microscopy and precise mechanical cutting. It is noted that IC packaging materials necessitate unique decapsulation approaches. For instance, plastic packages are typically decapsulated by chemical agents, such as concentrated sulfuric acid, while metal packages may be decapsulated by a laser-based technology, and ceramic packages sealed with an epoxy often require mechanical decapsulation by utilizing sophisticated cutting techniques. Improper decapsulation methods can lead to inaccurate outcomes of the failure analysis and inflated costs. So, identification of IC packaging materials is performed before its decapsulation. However, it is commonly implemented by human eyes, which brings a large number of human labors. Consequently, it is preferrable to develop an automatic method for the identification of IC packaging materials.

Some analysis methods have been widely used to analyze the materials of IC packages, involving X-ray fluorescence spectrometry, electrochemical impedance spectroscopy, differential scanning calorimetry, and nuclear magnetic resonance. These methods will meet several issues during the material identification of IC packages, although almost no errors emerge for their identifications. The first is that professional knowledge is required for analytic devices and material science. Second, a large amount of time is required for material analysis. Third, sampling inspection is usually utilized due to its low efficiency. Fourth, these analytic devices are significantly expensive. Nowadays, with the development of computer vision, automatic optical inspection has been widely employed in many industrial inspections due to its advantages of no contact, low economic cost, high efficiency, and a little professional knowledge. In particular, with the rapid development of deep learning, it has been successfully employed in the inspection of IC packaging. Hu et al. [3] designed a hierarchical convolutional neural network (CNN) to classify IC components, in which multiple deep features were efficiently merged in Convolutional Auto-Encoder (CAE) layers to reduce computational complexity. Cai et al. [4] designed a novel cascaded CNN for Surface Mount Technology (SMT) solder joint inspection, in which each layer adaptively learned the Region of Interest (ROI) of the IC solder joint image. Guo et al. [5] modified the YOLOv4 network with several strategies to identify electronic components in real time. One was to eliminate the downsampling stages within the feature pyramid framework. The other was to reformulate the loss function by the weighted sum of classification loss, confidence loss and localization loss. Ram et al. [6] developed an improved deep CNN to identify the defective wafers. They employed resampling to solve the problem of data imbalance and various optimizers to train the model. An et al. [7] designed a transformer-based classification approach to detect PCB defects, termed Label Robust and Patch Correlation Enhanced ViT (LPViT). Kao et al. [8] designed a multi-scale GAN model with an embedded transformer for surface defect inspection of IC packages, which incorporated a novel feature extraction scheme and a cross-scale feature fusion module into a multi-scale CNN encoder. Feng et al. [9] developed a transformer-based deep learning model for the classification of PCBs, which utilized masked region prediction to discern relationships among different areas in the features. Chen et al. [10] integrated a feature pyramid structure with the transformer as the backbone into YOLOv5 to effectively classify PCB components. 

Although these deep learning methods can easily implement the identification task by capturing the feature differences between different IC components or between the defect-free and defective IC packages, they may not be suitable for the identification of IC packaging materials. This is because IC components and their surface defects are quite different in their appearances, such as shapes and sizes. Comparatively, IC components made of different packaging materials usually exhibit highly similar overall appearances, necessitating distinction through a combination of features such as external structure, shape, and color. For example, plastic packages and metal packages typically have longer and fewer pins [11], with the latter also featuring identifiers that are laser-marked and embedded into the package [12], whereas ceramic packages have shorter and denser pins. Moreover, these comprehensive characteristics are possibly subtly different from each other. Thus, automatic identification of IC packaging materials is significant and challenging before IC decapsulation. 

In this paper, a hybrid deep learning framework is designed for automatic identification of IC packaging materials. To capture subtle appearance differences and some similar characteristics between IC packages with different materials, a CNN branch with two designed CNNs and a transformer branch with two transformers are constructed to focus on local/global features of IC packages with different materials, respectively. Since several characteristics of IC packages with various materials are related to each other, a bidirectional interaction strategy is designed to interactively transfer local/global features in channel and spatial dimensions between the CNN branch and the transformer branch. 

## 2. Methodology

### 2.1. Architecture of the Proposed Framework

**Figure 1:**
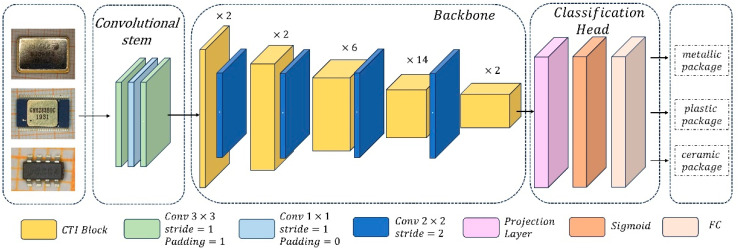
Architecture of the proposed hybrid deep learning framework. The input image dimensions are resized to 224 × 224. The convolutional stem expands the feature channels and makes global attention feasible. The backbone comprises five downsampling modules involving different CTI blocks ({2, 2, 6, 14, 2} successively) and a 2 × 2 convolutional layer with a stride of 2, serving as the downsampling module to halve the dimensions of the feature maps. The classification head yields a probability vector corresponding to the types of IC packaging materials.

Inspired by Efficientnet [13] and CoatNets [14], the proposed hybrid deep learning framework is designed as a feature pyramid architecture, which is composed of a convolutional stem, a feature-extracting backbone network, and a classification head. As shown in Figure 1, the convolutional stem is successively composed of a 3 × 3 convolutional layer, a 1 × 1 convolutional layer, and a 3 × 3 convolutional layer, which can expand the feature channels and make global attention feasible. The backbone is composed of several convolution–transformer interaction (CTI) blocks and four 2 × 2 convolutional layers, which are alternatively deployed in the backbone. The backbone is designed to comprehensively capture features from images of IC packages made of various materials. The classification head produces a probability vector that corresponds to different types of IC packaging materials, which is successively composed of a projection layer, a Sigmoid layer, and a fully connected (FC) layer. 

Specifically, the feature channels for the input electronic component image are increased from 3 to $C_{1}$ through three successive convolution layers in the convolutional stem. Then, the feature maps successively pass through five downsampling modules in the backbone, each of which involves {2, 2, 6, 14, 2} CTI blocks successively and a 2 × 2 convolutional layer with the stride of 2. That is, the feature maps are downsampled with the rates of $\left\{1,\;2,\;4,\;8,\;16\right\}$ through five downsampling modules. And their channels are progressively increased to $C_{5}$, that is, {64, 96, 192, 384, 768}. Next, in the classification head, the feature channels are expanded to 1280 after the feature maps pass through a projection layer and a Sigmoid layer. Finally, the identification is achieved when the activated feature maps pass through an FC layer in the classification head, which indicates metallic, plastic, or ceramic package. 

### 2.2. CTI Block

**Figure 2:**
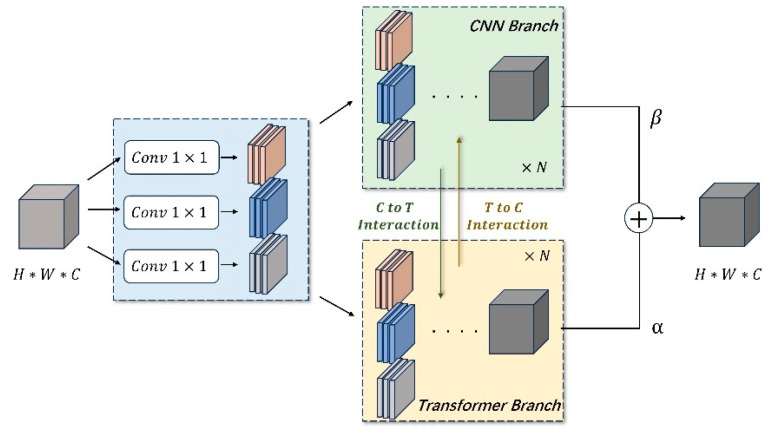
Sketch of the CTI block. The “C-to-T interaction” introduces the channel-wise features learned by the CNN into the transformer branch in a probabilistic fashion. Similarly, the global information learned by the transformer is integrated back into the CNN branch as probabilistic feature maps through the “T-to-C interaction”.

As illustrated in Figure 2, each CTI block is composed of three parallel 1 × 1 convolutional layers, a CNN branch, and a transformer branch. Specifically, the feature maps pass through three parallel 1 × 1 convolutional layers to generate the respective Q, K, and V feature maps [15], which can not only fulfill the requirements of the transformer branch but also add an extra feature extraction step for the CNN branch. Then, local/global features can be extracted by the CNN/transformer branches, respectively. It is noted that there is a bidirectional interaction between the two branches, which bidirectionally transfers local/global features to each other in channel and spatial dimensions. Finally, the feature maps output from two branches are adaptively fused through weighted summarization.

**Figure 3:**
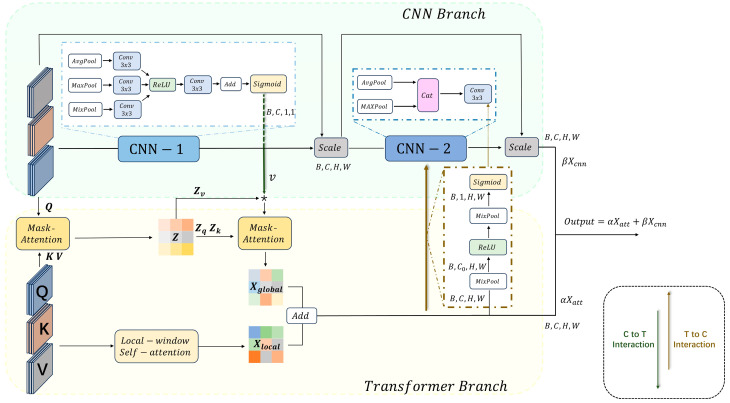
Detailed design of the two branches in the CTI block. The inter-channel feature maps extracted by CNN-1 are multiplied with (that is, the symbol * in Figure 3) the value matrix in global attention, compensating for the attention mechanism’s oversight of inter-channel information. The feature maps from the fusion of global and local attentions serve as the output for the transformer branch and as the input for T-to-C interaction, engaging in global information exchange with CNN-2.

As depicted in Figure 3, the transformer branch with a traditional transformer and a local-window self-attention integrates global information into local information through a broadcast mechanism. Concurrently, the CNN branch combines local and channel information by means of the residual design. The outputs from the two branches are then coalesced via a weighted summarization to produce the feature maps output by the CTI block. This structure harnesses the global receptive field of the transformer to capture wide-ranging dependencies and causes the CNN to focus on local spatial and channel features.

#### 2.2.1. Transformer Branch

Inspired by the “Adaptive Attention Span” [16,17], the attention is augmented by a masking function to enhance the transformer’s understanding of long-distance dependencies in the transformer branch, which can encourage each patch in the self-attention module to focus more on distant patches. 

In the standard attention mechanism, for a given patch m, similarities $b_{mn}$ are calculated between this patch and other patches at positions $n\;\in \;S_{m}$, where S denotes the contextual window for patch m, formulated as

(1) $$\begin{array}{c} b_{mn}=Q_{m}K_{n}^{T} \end{array}$$

where Q and K represent the Query and Key matrices, respectively. These are subjected to an activation function to obtain the attention weights $a_{mn}$ as

(2) $$\begin{array}{c} a_{mn}=softmax\left(b_{mn}\right)=\frac{\exp\left(b_{mn}\right)}{\sum_{i=m-s}^{m-1}\exp\left(b_{mi}\right)} \end{array}$$

**Figure 4:**
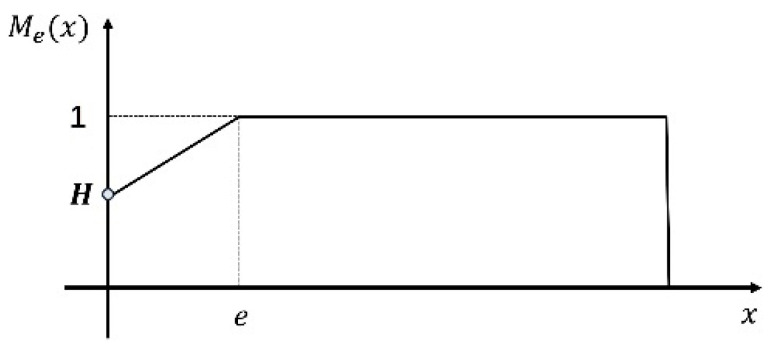
The masking function, which is designed to enhance the attention to focus on distant features.

Then, they are multiplied by the Value matrix to derive the attention vector $y_{m}$ as

(3) $$\begin{array}{c} y_{m}=\sum_{i=m-s}^{m-1}a_{mi}v_{i} \end{array}$$

where $v_{i}$ is the Value matrix for the i-th patch. Subsequently, a masking function $M_{e}\left(x\right)$ is introduced to the original attention to implement the “Adaptive Attention Span”, formulated as

(4) $$\begin{array}{c} M_{e}\left(x\right)=clamp\left(0,\frac{1}{e}\left(x-Hx+He\right),1\right) \end{array}$$

where H is a hyperparameter that governs the slope of the curve, which is empirically set to 0.5 in this work. e is differentiable and learnable. This masking function maps the distances between distinct patches into the interval of [0,1], which is graphically depicted in Figure 4.

Thus, a learnable token Z can be acquired by incorporating the masking function into the original attention, formulated as

(5) $$\begin{array}{c} Z=MAttention\left(Q,K,V\right)=\frac{M_{e}\left(m-n\right)\exp\left(b_{mn}\right)}{\sum_{i=m-s}^{m-1}M_{e}\left(m-i\right)\exp\left(b_{mi}\right)} \end{array}$$

The token Z can possess an expanded receptive field to learn more robust global dependencies. It is calculated from the QKV matrices only at the beginning of each downsampling module and will replace the *Q* matrix for propagation in the subsequent module, formulated as

(6) $$\begin{array}{c} \hat{Z}=MAttention\left(Z,K,V\right) \end{array}$$

Furthermore, to address the limitation of self-attention modeling capacity along the channel dimension, the convolution-to-transformer interaction (C-to-T interaction) is designed to transfer the v matrix in the local feature maps output by the CNN-1 in the CNN branch to $\hat{Z}$, formulated as

(7) $$\begin{array}{c} \hat{V}={\hat{Z}}_{v}v \end{array}$$

As indicated in (7), v can be considered as the inter-channel attention weights. Thus, the interacted Value matrix $\hat{V}$ is combined with the transferred *Q* and *K* matrices to construct the mask attention, i.e., global attention, formulated as

(8) $$\begin{array}{c} X_{global}=MAttention\left({\hat{Z}}_{q},{\hat{Z}}_{k},\hat{V}\right) \end{array}$$

To simultaneously capture local attention of distant dependencies, a local window self-attention [18] is combined with global attention to acquire the outputs $X_{att}$ of the transformer branch, formulated as

(9) $$\begin{array}{c} X_{att}=X_{local}+X_{global} \end{array}$$

where $X_{local}$ is the outputs of the local window self-attention, in which a 7 × 7 window is employed to balance performance with efficiency. 

#### 2.2.2. CNN Branch

*Q*, *K*, and *V* feature maps severally pass through three parallel pooling layers, each of which is followed by a 3 × 3 convolution layer. After activation by a ReLU layer, the activated feature maps pass through a 3 × 3 convolution layer, a pixel-wise summation, and a Sigmoid layer to produce a probability feature map v. This probability feature map with the dimensions $\left(B,C,1,1\right)$ reveals the correlational information of features in channel dimension, where B stands for batch size and C for the number of channels of the features maps. The acquisition of the probability feature map v can be formulated as

(10) $$\begin{array}{c} \left\{\begin{array}{c} v=\sigma \left(W_{2}\delta \left(W_{1}\left(z_{1}\right)\right)\right)+\sigma \left(W_{2}\delta \left(W_{1}\left(z_{2}\right)\right)\right)+\sigma \left(W_{2}\delta \left(W_{1}(z_{3})\right)\right)\\ z_{1}=AvgPool\left(F\right)\\ \begin{array}{c} z_{2}=MaxPool\left(F\right)\\ z_{3}=MixPool\left(F\right) \end{array} \end{array}\right. \end{array}$$

where F denotes the input feature maps. $W_{1}\in {\mathbb{R}}^{C_{0}\times C}$ and $W_{2}\in {\mathbb{R}}^{C\times C_{0}}$ represent convolutional kernels with $C_{0}<C$. σ() and δ() represent the Sigmoid/ReLU activation functions, respectively. To extract key features from the feature maps in the spatial dimension, a convolution-based spatial attention mechanism has been incorporated into the CNN-2 module, formulated as

(11) $$\begin{array}{c} \left\{\begin{array}{c} X_{cnn}=\sigma \left(f^{7\times 7}\left(\left[AvgPool\left(\hat{v}\right);MaxPool\left(\hat{v}\right);{\hat{x}}_{att}\right]\right)\right)\\ \hat{v}=F_{scale}\left(F,v\right) \end{array}\right. \end{array}$$

where ${\hat{x}}_{att}$ represents a single-channel feature map obtained by the dimensionality reduction of $X_{att}$ that is transferred from the transformer branch via the transformer-to-CNN interaction (T-to-C interaction). Thus, rich global information can be transferred into the CNN branch. $f^{7\times 7}$ signifies a 7 × 7 convolution to boast a larger receptive field compared with the 3 × 3 convolution, which can place a greater focus on the spatial dimension. $X_{cnn}$ denotes the output of the CNN branch.

#### 2.2.3. Bidirectional Interaction

Typically, a self-attention mechanism employs a weight-sharing strategy across the channels of features, with a focus on the weights in the spatial dimensions, which tends to overlook inter-channel dependencies for weak dimensional modeling. This problem can be solved by sharing channel-related weights [19]. That is, in our designed framework, the CNN branch can offer “clues” to the transformer branch in the form of channel-wise attention weights. Conversely, the outputs of the transformer branch can supplement the CNN branch with spatial-attention clues. Thus, this bidirectional interactive design can enhance the modeling capacities of the framework in the channel-spatial dimensions. 

As mentioned in Section 2.2.1 and Section 2.2.2, a bidirectional interaction interactively transfers global/local feature maps between the two branches, involving a C-to-T interaction for local information transmission and a T-to-C interaction for global information transmission. As indicated in (7), the C-to-T interaction transfers local feature information *v* into the transformer branch to enrich probability distribution of local features along the channel dimension. Similarly, the T-to-C interaction transfers global feature information $X_{att}$ acquired by the transformer branch into the CNN branch to enrich the probability distribution of global features along the spatial dimension, formulated as

(12) $$\begin{array}{c} {\hat{x}}_{att}=\sigma \left(MixPool\left(\delta \left(MixPool\left(X_{att}\right)\right)\right)\right) \end{array}$$

where *MixPool*() [20] denotes an adaptive selection of maxpooling or average pooling.

## 3. Experiments and Discussion

### 3.1. Dataset and Experimental Environments

**Figure 5:**
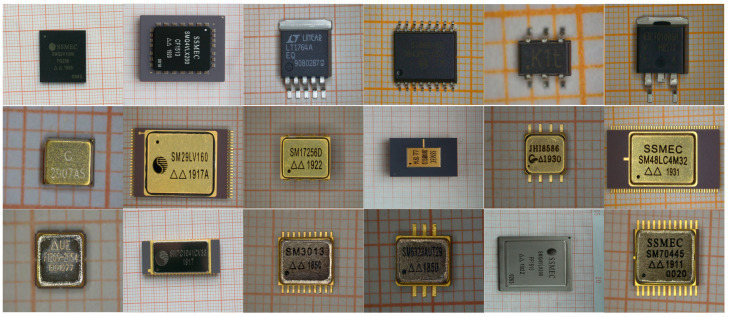
Some examples of IC packages with different materials. The first/second/third rows refer to IC packages with plastic/ceramic/metallic materials, correspondingly.

All the images for IC packages with different materials were acquired by the industrial microscope (LV150N/LV100ND) in China Electronic Product Reliability and Environmental Testing Research Institute (Guangzhou, China). During the image acquisition, the microscope utilized the LV-S64 6 × 4 stage (Stroke: 150 × 100 mm with glass plate, ESD compatible) to place the electronic components, the LV-TI3 trinocular eyepiece tube ESD (Erected image, FOV: 22/25) as the eyepiece tube, the LV-NU5 U5 ESD as the objective revolver, and the LV-UEPI-N as the built-in reflected light illuminator. All the parameters of the microscope were elaborately configured to achieve sufficiently clear visibility of the markings on the IC packages. Then, 945 IC packaging images of 420 × 400 to 1280 × 960 pixels were acquired for three types of packaging materials such as metallic, ceramic, and plastic packages. Specifically, IC packages with each type of material involve 315 samples, some examples of which are illustrated in Figure 5. Different from pixel-level annotations, only category annotations are required for this study. Since all the acquired images were stored in three folders according to packaging materials during the image acquisition, we could directly utilize the folder names as the image labels, which indicated that no additional labeling task was required.

All the acquired images were rotated by ±30° and ±15° for data augmentation. Thus, 4725 images were utilized to construct the experimental dataset for the identification of IC packaging materials, which were all resized to 224 × 224 pixels to standardize the input for the proposed model. The dataset was divided into training, validation, and test sets in a completely random method at a ratio of 6:3:1, which was illustrated in Table 1.

In this study, all the weights were initialized as a Gaussian distribution with a mean of 0 and a variance of 0.01. The learning rate was set to $10^{-4}$, and the Adam was chosen as the optimizer. Momentum and weight decay were set to 0.997 and 5 × 10^−5^, respectively. The loss function used cross-entropy loss. All the experiments were conducted on a computer equipped with an Intel(R) Core(TM) i7-9750H CPU @ 2.60 GHz and an Nvidia GeForce RTX 2080 12 GB GPU. The CPU was produced by Intel Corporation, located in Santa Clara, CA, USA, and the GPU was sourced from NVIDIA Corporation situated in Santa Clara, CA, USA. Both of the hardware were purchased in Guangzhou, China. Several commonly used metrics were employed for evaluation, involving accuracy, precision, recall, and F1-score [21].

### 3.2. Comparisons with Other Deep Learning Models

To the best of our knowledge, no studies have focused on the identification of IC packaging materials. Thus, to validate the proposed framework, it is compared with some state-of-the-art (SOTA) deep learning models for image classification, involving five hybrid models (MixFormer [22], Acmix [15], CoAtNet_4 [14], SMT [23], and FastVit_V3 [24]), four CNN models (ConvNeXt_S [25], FasterNet [26], ResNet_152 [27], and Wafer classification [6]), and three transformer models (Swin-Transformer_l [28], ITPN [29], and LPViT [7]). It is noted that all the models were equipped with the same classification head and were retrained on our training set. The experimental results are summarized in Table 2.

As indicated in Table 2, the three transformer models perform quite differently in identifying IC packaging materials. Specifically, the ITPN [29] achieves the worst identification performance with the second heaviest computational complexity among all the models, with the performance of 37.27% F1-score, 40.90% accuracy, and 59 G FLOPs. This means that the ITPN is of low efficiency in model inference and prediction. This is because its multi-layer feature pyramid structure excessively focuses on ultra-small objects, which possibly neglects distant dependencies existing in IC packages with different materials. Since the LPViT [7] directly employs the traditional transformer to extract global features from the images, it will neglect the local information of the IC package image, which involves some important hints to distinguish subtle differences between the IC packages with different materials. Thus, it also achieves a bad classification performance of 66.54% F1-score and 73.57% accuracy. The Swin Transformer [28] almost achieves the best identification performance among all the single models, with the performance of 82.13% F1-score and 82.21% accuracy. This can be attributed to its shift-window mechanism, which allows each local window to learn feature information from surrounding windows to effectively combine local details with global information. Also, this shift-window mechanism decreases the computational complexity while increasing the model size compared to the traditional transformer, with the parameter size of 39 M and 34 G FLOPs.

Although the CNN can capture local information from the image, the ability of local information extraction increases with the increase in the network depth. That is, if the network depth is not large enough, the CNN cannot efficiently extract local information at a high probability, which will significantly degrade the classification ability of the network. So, the Wafer classification [6] performs the second worst identification and has the lowest parameter size among all the methods due to its very shallow network architecture, with the performance of 54.24% F1-score, 59.22% accuracy, and 10 M parameters. Another three CNN models, namely ConvNeXt_S [25], FasterNet [26], and ResNet_152 [27], perform fairly good identification tasks for IC packages with different materials, even better than some hybrid models. This is because they can capture some subtle differences that exist in the IC packages with different materials, which is beneficial for identification. In particular, the FasterNet combines partial convolutions and point-wise convolutions [30] to speed up the model inference with a good model prediction, with an identification performance of 80.09% F-score and 83.57% accuracy, and a computational complexity of 4.4 G FLOPs. Since the ConvNeXt elaboratively integrates some previously published effective modules to fully mine the potential of the CNN framework to classification, it costs the most computational resources (61 G FLOPs) to establish a fair classification model for IC packaging material identification with a performance of 81.48% F1-score and 81.56% accuracy.

Since the CNN is good at capturing local features in the image, while the transformer is adept at understanding wide-range dependencies, the combination of the CNN and transformer can ideally result in better identification tasks being performed than most of the single models [31] (CNNs or transformers). As indicated in Table 1, three hybrid models (Acmix [15], MixFormer [22], and Ours) are the three best models for IC packaging material identification among all the models. However, the other three hybrid models (SMT [23], FastVit_V3 [24], and CoAtNet_4 [14]) do not exhibit superiority to single models. The two entirely different identification results can be contributed to their different hybrid schemes for the combination of CNNs and transformers. The former three hybrid models integrate CNNs and transformers into the total framework in a parallel mode, while the latter three hybrid ones sequentially cascade CNNs and transformer blocks. In particular, the FastVit_V3 achieves the second worst identification performance of 62.71% F1-score and 61.76% accuracy. This is because, except for the sequential cascade, it substitutes RepMixer and channel-wise and point-wise channel convolutions for skip connection and dense convolutions, respectively, to decrease its parameter size and computational complexity, which also to a great extent degrades its prediction performance.

Our designed framework achieves the best identification performance for IC packages with different materials at a reasonable inference speed and an acceptable model size. Its identification performance of 96.16% F1-score and 97.92% accuracy demonstrates its potential in real applications, and for which there are several reasons. One is its parallel combination of CNNs and transformers, which can simultaneously capture local and global features from the IC packaging image. Second, the elaboratively designed bidirectional interaction can integrate local information into the transformer branch and global information into the CNN branch, which can simultaneously capture local/global features in channel and spatial dimensions. Third, the combination of local-window self-attention and mask attention can together capture global features in global and local attentions, which can further improve the global feature representations for IC packaging images.

### 3.3. Ablation Experiment

We conducted an ablation experiment to validate the two branches, the bidirectional interaction, and weighted summarization, as illustrated in Table 3.

As indicated in Table 3, the simple combination of CNN and transformer branches performs a better identification of IC packaging materials than only a single branch, with the classification performance of 79.35% F1-score and 81.56% accuracy. This demonstrates that CNN and transformer branches both contribute to the model since they provide local/global feature information, respectively. When a one-way interaction is deployed between the two branches, the identification performance is more or less improved. In particular, if only the C-to-T interaction is incorporated into the proposed framework to transmit channel information provided by the CNN branch to the transformer branch, the classification performance significantly improves to more than 7% F1-score and 8% accuracy compared to the two-branch model without any interactions. This indicates that the two information interactions are beneficial for the fusion of global and local features in the IC packaging image, which can potentially provide deep subtle differences between different IC packaging materials. Thus, when the information interactions are bidirectional for the two branches, the model with a bidirectional interaction performs much better than those with a one-way interaction, which indicates that this bidirectional interaction can interactively transmit local/global features between the two branches to enhance their abilities of feature extraction. Since the two branches contribute differently to the designed framework, the model with weighted summarization of the branches (96.16% F1-score, 97.92% accuracy) improves the F1-score and the accuracy by almost 3% and 4%, respectively, compared to that with fixed fusion of the branches (93.19% F1-score, 94.08% accuracy).

### 3.4. Application for the Other Classification Task

To assess the application ability of our proposed model for the other specific task, we constructed an electronic component dataset from the Findchips website [32] for another classification task. The Findchips website is a powerful search engine that aggregates and normalizes data from the leading electronic part distributors in the electronic component market, which involves the attribute data, models, and schematics of the electronic components. There are 35 categories of electronic components on this website, such as resistor, capacitor, connector, transistor, and diode, each of which involves more than 1000 images.

To construct the Findchips dataset for the assessment of our proposed model, 200 images were randomly selected from each category on the Findchips website. Thus, a total of 7000 electronic component images were involved in the Findchips dataset, which were divided into training, validation, and test sets in a completely random method at a ratio of 6:3:1. Our proposed framework with the parameters configured in Section 3.1 was retrained, validated, and tested on the Findchips dataset. As illustrated in Table 4, the proposed model can also implement the other classification-specific task, although it is originally designed to identify IC packaging materials. However, since it is not specifically designed for this 35-category classification, its classification performance decreases to an 83.25% F1-score, compared to a 94.93% F1-score for 3-category IC packaging materials. This reveals the common shortcoming of deep learning, which is that task-specific deep learning models can be directly utilized but do not function well in the other tasks.

### 3.5. Repeated Experiments

To verify the reproductivity of the proposed framework, 10 repeated experiments were conducted on the augmented data with the sizes of 224 × 224 pixels, that is, 4725 images of IC packages with different materials. In each experiment, these images were randomly divided into training, validation, and test sets at a ratio of 6:3:1. This indicates that the samples in the three sets are different for each experiment.

As indicated in Table 5, the proposed framework achieves different identification performances for different experiments due to their different sample distributions. However, this difference is relatively small, with the largest and the smallest F1-scores being 96.16% and 91.98%, and the largest and the smallest accuracies being 97.92% and 94.83%, correspondingly. The classification performances of most of the repeated experiments approach the average metrics of 94.84% for precision, 95.23% for recall, 94.93% for F1-score, and 96.52% for accuracy, which demonstrates that the proposed framework has a fairly a good reproductivity ability.

## 4. Conclusions

In micro- and nanomanufacturing, accurate identification of IC packaging materials is crucial for quality control, since it is significant for packaging decapsulation. Misidentification may destroy the internal structures of IC components and chips during its decapsulation.

To identify IC packaging materials, a hybrid deep learning framework is designed in this paper, which adaptively integrates CNNs and transformers into an entire framework. The framework features two branches (i.e., CNN and transformer branches) with a bidirectional interaction to effectively capture local/global features in the IC packaging image to deeply mine the differences of IC packaging materials, which are validated by the ablation experiment. Comparative experiments indicate that the designed hybrid framework can better identify three types of IC packaging materials for the similar appearances of IC packages than the existing deep learning methods, with the performance of 94.84% precision, 95.23% recall, 94.93% F1-score, and 96.52% accuracy.

Although several CTI blocks improve the identification ability of the proposed model, they bring additional computational resources and computational burden due to their complex network design. In particular, the traditional vision transformer architecture in the CTI block further aggravates this issue. This limits the application of the proposed method in the assembly lines of real industries, since time cost and economic cost are sensitive in these scenarios. In the future, we will study the lightweight implementation of the proposed model to decrease the network size and speed up the network inference, such as the substitution of the mobile vision transformer for the traditional one. Since all the electronic components are provided by real industries, the experimental data acquired in the lab are prone to industrial ones, which indicates that the proposed model can be deployed in real industries for online identification in the future. However, the operation environments in real industries are quite different from lab ones, so the model will be retrained to adapt the data acquired in real industrial environments. If these issues are solved, our proposed model can be deployed in the assembly lines of real industries to automatically screen IC packages with different materials in the future, which can improve the efficiency of purpose-specific surface mounting, decapping, or recycling.

## Figures and Tables

**Table 1:** Statistics of the dataset.

Dataset	Plastic	Ceramic	Metallic	Total
Training	911	951	963	2835
Validation	455	475	481	1401
Test	209	149	131	489
Total	1575	1575	1575	4725

**Table 2:** Comparisons of different deep learning models for identification of IC packaging materials.

Method	Precision (%)	Recall (%)	F1-Score (%)	Accuracy (%)	FPS	Params (M)	FLOPs (G)
ITPN [29]	76.45	47.04	37.27	40.90	1.02	29	59
Swin-Transformer_l [28]	82.82	84.42	82.13	82.21	1.36	39	34
LPViT [7]	74.34	66.81	66.54	73.57	1.12	28	41
ResNet_152 [27]	83.84	80.70	78.48	79.35	2.54	32	12
FasterNet [26]	74.34	86.81	80.09	83.57	1.41	25	4.4
ConvNeXt_S [25]	84.53	83.49	81.48	81.56	1.53	35	61
Wafer classification [6]	64.16	59.34	54.24	59.22	3.31	10	18
SMT [23]	84.18	83.83	81.27	81.18	1.76	30	41
FastVit_V3 [24]	79.70	68.02	62.71	61.76	2.28	22	27
CoAtNet_4 [14]	75.51	74.45	70.98	71.78	1.59	38	55
ACmix [15]	89.75	83.12	86.39	88.39	1.62	29	4.5
MixFormer [22]	91.19	86.09	88.34	90.67	1.47	56	9.6
Ours	96.12	96.26	96.16	97.92	1.99	44	23

**Table 3:** Ablation experiment.

Transformer Branch	CNN Branch	C-to-T Interaction	T-to-C Interaction	Weighted Summarization	F1-Score (%)	Accuracy (%)
✓					73.99	79.40
	✓				78.39	81.39
✓	✓				79.35	81.56
✓	✓	✓			86.69	90.39
✓	✓		✓		79.83	87.56
✓	✓	✓	✓		93.19	94.08
✓	✓	✓	✓	✓	96.16	97.92

**Table 4:** Classification results for the Findchips dataset via the proposed model.

Method	Precision (%)	Recall (%)	F1-Score (%)	Accuracy (%)
Ours-1	90.85	76.83	83.25	87.61

**Table 5:** Ten repeated experiments for the proposed framework.

Index	Precision (%)	Recall (%)	F1-Score (%)	Accuracy (%)
1	95.94	95.98	95.99	96.55
2	94.15	95.88	94.99	96.41
3	94.95	95.78	95.38	96.58
4	95.35	94.97	95.18	96.66
5	93.79	94.83	94.43	95.97
6	95.41	95.79	95.58	95.31
7	96.12	96.26	96.16	97.92
8	94.99	95.52	95.39	96.96
9	91.96	91.44	91.98	94.96
10	95.74	95.85	95.81	97.89
Average	94.84	95.23	94.93	96.52

## Data Availability

The original contributions presented in the study are included in the article, further inquiries can be directed to the corresponding author.
